# Effects of Tai-Chi and Running Exercises on Cardiorespiratory Fitness and Biomarkers in Sedentary Middle-Aged Males: A 24-Week Supervised Training Study

**DOI:** 10.3390/biology11030375

**Published:** 2022-02-26

**Authors:** Yi Wang, Xian Guo, Liangchao Liu, Minhao Xie, Wing-Kai Lam

**Affiliations:** 1 Department of Physical Education, Renmin University of China, Beijing 100872, China; 2 Sports and Social Development Research Center, Renmin University of China, Beijing 100872, China; 3 School of Life Science and Technology, Harbin Institute of Technology, Harbin 150001, China; 4 Sport Science School, Beijing Sport University, Beijing 100084, China; guoxian@bsu.edu.cn; 5 Physical Education Department, University of International Business and Economics, Beijing 100029, China; 02289@uibe.edu.cn; 6 National Institute of Sports Medicine, Beijing 100062, China; mhaoxie@126.com; 7 Sports Information and External Affairs Centre, Hong Kong Sports Institute, Sha Tin, Hong Kong

**Keywords:** home-based exercises, heart rate, aerobic exercise, cardio-metabolic biomarkers

## Abstract

**Simple Summary:**

Tai-Chi is a popular indoor-based exercise that could induce several health-related benefits, but its benefits for middle-aged adults are unclear. Tai-Chi exercise can equally improve cardiorespiratory fitness, resting health rate, blood pressure and lean mass as a moderate-intensity running exercise. Running exercises appears to have further benefits in reducing the blood lipid level. The results from this study can provide insights into various types of exercises on cardiorespiratory fitness and biomarkers in the middle-aged population.

**Abstract:**

This study examined the effectiveness of Tai-Chi and running exercises on cardiorespiratory fitness and biomarkers in sedentary middle-aged adults under 24 weeks of supervised training. Methods Thirty-six healthy middle-aged adults (55.6 ± 5.3 yr) were randomly assigned into Tai-Chi, running and control groups. During a 24-week training period, the Tai-Chi and running groups were asked to perform exercises for 60 min/day and 5 days/week, which were supervised by Tai-Chi and running instructors throughout. Resting heart rate, lean mass, blood pressure and blood lipids were measured, and cardiorespiratory fitness (VO_2max_, V_max_ and Peak heart rate) was assessed at the baseline and the 12- and 24-week interventions. Results Compared to the no-exercise control group, both the Tai-Chi and running groups significantly decreased resting heart rate, diastolic blood pressure and cardiorespiratory fitness and increased lean mass across the training session (*p* < 0.05). Compared to the Tai-Chi group, the running group showed greater improvement in VO_2max_ and V_max_ (*p* < 0.05) and reduced triglyceride and low-density lipoprotein cholesterol (*p* < 0.05). Conclusion Both Tai-Chi and running exercise showed beneficial effects on cardiorespiratory fitness and enhanced health-related outcomes in middle-aged adults. Although Tai-Chi exercises were less effective in VO_2max_ than running, Tai-Chi may be considered as a plausible alternative to running exercises that can be achieved in the indoor-based setting.

## 1. Introduction

Cardiorespiratory fitness refers to the functional capacity of the circulatory and respiratory systems to supply oxygen for the energy production needed during physical activity. It is considered to be an effective and sensitive predictor of cardiovascular disease and other chronic diseases [1,2,3]. A meta-analysis showed that cardiorespiratory fitness had a negative correlation with coronary heart diseases and all-cause mortality in the general population [3]. The risks of coronary heart disease and all-cause mortality were estimated to decrease by 13–15% of exercise capacity per metabolic equivalent (MET) (1 MET is equal to 3.5 mL/kg/min) [3].

There are several exercise recommendations to improve cardiorespiratory fitness in the literature [4,5]. The optimal exercise intensity of individuals depends on the functions of the pulmonary, cardiovascular and musculoskeletal systems. The type, intensity, frequency, and duration of exercise regimens are the most important determinants to improve cardiorespiratory fitness. Moreover, individual cardiorespiratory fitness is associated with the maximal oxygen uptake (VO_2max_). According to the American College of Sports Medicine (ACSM) recommendation, the exercise intensity is prescribed between 64% and 90% of the maximal heart rate (HR_max_) to improve the myocardial adaptability [6]. Furthermore, Hickson et al. [7] suggested exercising more than four times per week is optimal as it was shown to have greater VO_2max_ improvement than exercises with fewer exercise sessions. 

To date, common aerobic exercises such as swimming, jogging, and running are recommended as they are easy to assess and also improve cardiorespiratory fitness. While Tai-Chi is a popular form of exercise in Asian countries, as well as worldwide [8,9], little is known about its effect on cardiorespiratory fitness. Tai-Chi is a mind–body exercise, which is proposed to integrate all physical and spiritual elements to gently move “qi” (vital energy) throughout the body. Tai-Chi has been shown to induce health related benefits, which include improved aerobic capacity and body composition [6], reduced risk potentials of chronic diseases [10,11,12], reduced blood pressure [13], and better dynamic balance [14,15] and muscular strength [14,16]. Furthermore, regular Tai-Chi exercises improved VO_2max_ in sedentary older adults [17,18]. 

However, investigations have been predominantly focused on older adults, so the results may not be directly applied on middle-aged adults who have clear differences in physical condition and movement patterns. Physical activity research on the middle-aged population would be of great benefit for the quality of life and fitness in the older age population. Moreover, most of the studies comparing different types of exercises did not strictly control the frequency/duration and nutrient intake. It is questionable if Tai-Chi would be more beneficial on cardiorespiratory fitness than the commonest prescribed exercises, such as running, under the same amount of exercise (frequency/duration and nutrient intake) in the middle-aged population. Hence, the aim of this study was to compare the effects of Tai-Chi and running exercises on cardiorespiratory fitness and cardiometabolic biomarkers under 24-week supervised training. The results from this study can provide insights and application guidelines of exercises for the healthy, sedentary middle-aged population. 

## 2. Materials and Methods

### 2.1. Participants

Healthy sedentary middle-aged male adults were recruited from a local community. All of them were reported to have fewer than 1000 steps each day and no regular exercise habits. An Actigraph accelerometer (ActiGraph wGT3x+, ActiGraph, Pensacola, FL, USA) on the right hip was used over consecutive days to confirm all participants had fewer than 1000 steps per day on average (Table 1). They were excluded if they were smokers, they had a body mass index (BMI) greater than 30 kg/m^2^, or they had coronary artery disease, anemia or other cardiovascular diseases. A total of 45 male middle-aged adults were recruited with an incentive of USD 350 for this 24-week supervised exercise study. The participants were randomly assigned into each of three exercise groups (Tai-Chi, running and no exercise control). There were nine participants who withdrew from this study due to sickness (*n* = 3) and unable to fit their personal schedule (*n* = 6). The others completed the entire 24-week exercise session. Eventually, a total of 36 participants completed the entire 24-week exercise sessions (120 days) for the Tai-Chi (*n* = 10), running (*n* = 14) and control (*n* = 12) groups. The demographic characteristics of the participants are shown in Table 1. The body mass and lean mass were measured with the InBody body composition analyzer (InBody 230, InBody, Seoul, Korea). There were no significant differences between exercise groups in age, height, weight, BMI, lean mass and self-reported physical activity level (with International Physical Activity Questionnaire, IPAQ) [19] before exercise interventions (baseline). The self-reported questionnaire consisted of five physical activity categories including work, transportation, housework, leisure time and sedentary time for the last 7 days and was completed by each participant. To objectively determine the duration of the reported physical activity, the participants were instructed to wear the Actigraph accelerometer (ActiGraph wGT3x+, ActiGraph, Pensacola, FL, USA) on the right hip for the entire day and overnight (except when bathing) over 7 consecutive days to track the physical activity. Based on the time adjusted with the Actigraph data, the overall energy expenditure of physical activity was calculated using the established IPAQ formula (i.e., cardiorespiratory endurance of health-related physical fitness testing and analysis) [19]. Ethical approval was given by the medical ethics committee of Beijing Sport University Sport Science Ethical Committee (2014037). All participants provided their informed consents before any measurements. 

### 2.2. Exercise Intervention

**Figure 1:**
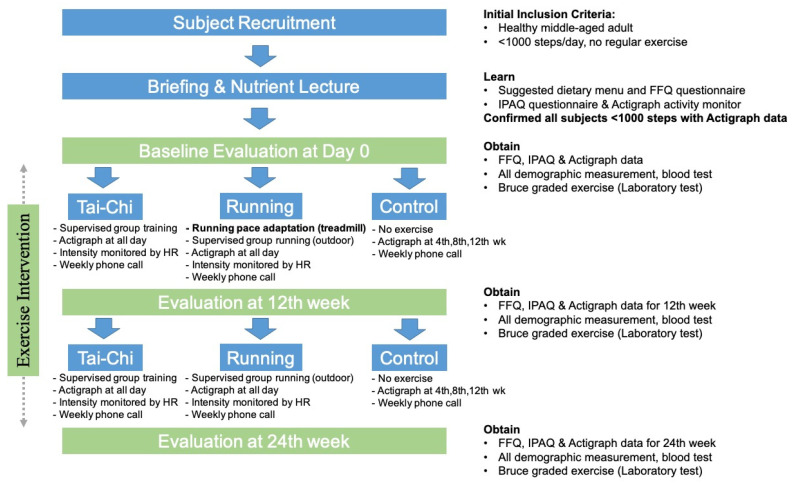
Experimental workflow for Tai-Chi, running and control groups.

Participants were randomly assigned into Tai-Chi, running and control groups in this study. The HR_max_ was calculated by 207–0.7 * year for males [19]. All participants attended the briefing and nutrient lectures to learn how to record their daily nutrient intake using Food Frequency Questionnaires (FFQ) (Figure 1) [20]. The participants in the exercise group (Tai-Chi and running) were prescribed with a suggested daily recipe of 2800 kcal and the control participants with 2700 kcal throughout. Such dietary intake designs were used to minimize the confounding effect due to the energy expenditure between the exercise and no-exercise control groups. The ratio between carbohydrates, fats and proteins was set at 55–30–15% of calories in all recipes, which was referenced from a book on Chinese food composition for our participants [21]. All of the participants were told not to have any great change in dietary uptake habit across the entire 24-week period (Figure 1). They were also reminded of how to wear the Actigraph activity monitor and heart rate monitoring watch (Galaxy, Samsung, Seoul, Korea). All of the participants were instructed to perform graded treadmill running to determine the VO_2max_ for individual participants at baseline, based on the Bruce protocol (Figure 1, [22]). The participants also indicated their Rating of Perceived Exertion (RPE) with the Borg scale (6–20) at 50–60% and close to VO_2max_, respectively. The RPE level at close to VO_2max_ was used as a reference to determine VO_2max_. The 50% and 60% of the VO_2max_ were then used to determine the corresponding heart rate and running speed on a treadmill. The 50–60% of the VO_2max_ level corresponded approximately to 66–74% of the maximal heart rate (i.e., 110–130 b/min) for Tai-Chi, running and control group participants. The 50–60% of the VO_2max_ level was also equivalent to about the running speed of 5.8–7.2km/h and step frequency of 150–158 step/min used for the running group.

Participants in both Tai-Chi and running groups exercised outdoor between 7 and 9 am, 60 min per day and 5 days per week, for 24 weeks under supervised training. The total exercise duration was 7200 min/120 h (60 × 5 × 24 min) over the 24 weeks. The participants were required to wear the heart rate monitor watch (Galaxy, Samsung, South Korea) and Actigraph activity monitor during the entire experiment, including exercises, non-exercise activities and sleeping. Before the intervention, participants in the Tai-Chi group attended a 2 h class once per week for four weeks to learn the Chen style of Tai-Chi and each movement step was executed in about 60–65 steps/min, which has moderate intensity around 66–74% HR_max_, specified in our pilot study. Participants acquired a full set of Tai-Chi techniques including breathing, balance, flexibility, concentration, calming, and stress-reduction techniques. During the early learning sessions, the instructors taught 4 movements per session, and this accumulated as sessions increased. All participants learned all movements of Chen-style Tai-Chi within the first four weeks. After that, the participants repeatedly performed the same Tai-Chi movements under the supervision of the same Tai-Chi instructor. Prior to each exercise session, participants were asked to perform a 20 min warm-up and activated the alarm of the heart rate monitor, which provided haptic vibration feedback on the wrist if the participant’s heart rate did not lie within the range of 66–74% HR_max_ (i.e., 110–130 b/min) at any time during exercise. The instructors monitored the performance of each participant and confirmed that all participants had good compliance in their sessions. 

Participants in the running group performed running exercise at 66–74% HR_max_ (i.e., 110–130 b/min), which corresponds to a running speed of 5.8–7.2 km/h and a step frequency of 150–158 step/min. The range of heart rate and running speed/step frequency were predetermined with the 50–60% of the VO_2max_ level before the start of the exercise intervention, which has previously been used in earlier studies [19,22]. Since all of the participants were sedentary and did not have exercise habits, the participants were allowed to familiarize themselves with the running speed and step frequency on the treadmill prior to the first running session (Figure 1). At each running session (outdoor), participants firstly performed a 20 min warm-up and activated the heart rate monitor alarm to control their heart rate between 66−74% HR_max_ (i.e., 110–130 b/min) during the running sessions. Two experienced running instructors served as the pacemakers for the speed of 5.8 km/h and 7.2 km/h to ensure all participants were running with the same intensity throughout the 24-week experiment. Participants provided a training diary to record their running mileage, which was monitored, and they were reminded not to have a significant change in their non-exercise activities weekly by telephone calls. The training diary was collected after the final running session.

Participants in the control group were asked to maintain their physical activity level and nutrient uptake as they acquired in the briefing and nutrient lectures (Figure 1). The participants were required to wear the Actigraph tracker on their right hip over the consecutive 7 days except during bathing every 4 weeks (i.e., 4th, 8th, 12th, 16th, 20th and 24th week) as a reminder to keep their weekly physical activities constant. 

For the Tai-Chi, running and control groups, the participants were invited to come back to our laboratory to complete the activity questionnaire (IPAQ) together with the reference of their weekly Actigraph data to determine the overall physical activity level at baseline (0 week), in the 12th week and in the 24th week. In all three groups, we used the Food Frequency Questionnaire (FFQ) [20] to measure their dietary intake status before and during the intervention. The weekly telephone calls were given to all participants to ensure they were keeping their physical activity and dietary intake constant.

### 2.3. Evaluation Tasks

At baseline, and after 12 and 24 weeks, all participants visited the laboratory. The participants were asked to complete the activity questionnaire (IPAQ) and foot frequency questionnaire (FFQ) as well as measure their body composition. Their cardiorespiratory fitness and biomarkers were assessed by the same exercise physiologist and registered nurse. After participants had sat quietly for 10 min, their resting heart rate (HR) was measured with a polar chest-belt monitor (Polar 5 pulse, Polar, Kempele, Finland) and blood pressure, including systolic blood pressure (SBP) and diastolic blood pressure (DBP), was measured with an electronic sphygmomanometer (SunTech Tango^+^, SunTech Medical, Morrisville, NC, USA).

Blood samples were obtained between 7:00 am and 8:00 am after overnight fasting. They were obtained from an antecubital vein and collected in a BD Vacutainer plasma tube (Becton, Dickinson and Company, Franklin Lakes, NJ, USA). Plasma was isolated by centrifugation at 3500 rpm for 10 min and frozen at −80 °C within two hours of collection. Analyses were subsequently completed within three months of collection. Five metabolic indicators, including plasma triglycerides (TG), total cholesterol (TC), high and low-density lipoprotein cholesterols (HDL-C and LDL-C), and fasting blood glucose (FBG), were determined.

Graded exercise test design was used to assess the cardiorespiratory fitness, including VO_2max_ and maximum aerobic speed (V_max_), which is in accordance with the Bruce protocol [22]. V_max_ is determined as the maximum termination velocity when the participants finished their graded exercise tests. We asked the participants to give their RPE throughout the graded exercise test. The RPE at the 50–60% VO_2max_ level was extracted to allow comparisons among the Tai-Chi, running and control groups, as this VO_2max_ range was used to set the exercise intensity for our running and exercise groups. Participants were instructed to avoid any intake of medicine or alcohol and not to perform any vigorous physical activity for 12 h before the running test. During the graded exercise tests, peak heart rate (HR_peak_) and blood pressure were monitored. The VO_2max_ tests were analyzed breath-by-breath and by calculating the mean values from expired air at 30 s intervals using a metabolic measurement device (Cortex Metalyzer-III, Cortex, Leipzig, Germany).

### 2.4. Data Analysis

All statistical analyses were performed using SPSS 17.0. The data normality was examined with Shapiro–Wilk tests for each of the dependent variables and the findings indicated that the data in all variables were normally distributed, except for HDL, LDL and FBG. The Geisser’s epsilon adjustment was applied when the Mauchley test found a violation in the sphericity assumption. Levene’s test was also used to assess the equality of variances between different groups. Two-way (3 exercise × 3 time) factorial ANOVA with repeated measures was used to determine the interaction and main effects for each of the tested variables. When a statistically significant difference was observed, a Bonferroni post hoc comparison (or Dunnett T3 test if the assumption of normal data or equality of variances was violated) was performed. The alpha was set at 0.05 for all analyses. 

## 3. Results

### 3.1. Physical Activity and Nutrient Uptakes

There was a significant interaction between group and time in average number of steps per day (*p* < 0.001) and self-reported physical activity (*p* < 0.001, Table 2). The simple main effect revealed that the Tai-Chi and running groups had a higher average number of steps per day, which was found at 12 weeks and 24 weeks compared with the baseline (*p* < 0.001), while the control group remained robust across the training time (*p* > 0.05). The group effect indicated a higher number of steps per day in Tai-Chi and running than the control (*p* < 0.001), while no significant group difference for the self-reported physical activity was found (*p* > 0.05).

For nutrient intakes, significant interactions were determined in total dietary carbohydrate, protein and fat intakes (*p* < 0.001, Table 2). The simple main effect indicated that both Tai-Chi and running groups demonstrated higher total dietary carbohydrate, protein and fat intakes in 12 weeks and 24 weeks compared to baseline (*p* < 0.05), while levels remained unchanged across training time in the control group (*p* > 0.05). 

### 3.2. Demographic Characteristics

There was a significant interaction between group and time in resting HR, body mass and lean mass (Table 3, *p* < 0.005). The simple main analysis indicated that the magnitude of the resting HR was decreased by 13.8 ± 2.3 b/min (*p* < 0.001) in the Tai-Chi group and −3.7 ± 1.5 b/min, (*p* = 0.021) in the running group after the 24-week intervention. The analysis also revealed that lean mass increased over the 24-week training in the running and Tai-Chi groups (*p* < 0.05), but not changed in the control group. 

### 3.3. Blood Pressure

The systolic blood pressure (SBP) and diastolic blood pressure (DBP) are shown in Table 3. There was an interaction between group and time in DBP (*p* = 0.006), but not in SBP (*p* > 0.05). After 24-week intervention, the Tai-Chi group (7.5 ± 3.2 mmHg, *p* = 0.029) and running group (8.0 ± 2.2 mmHg, *p* = 0.001) DBP was significantly lower than at baseline. 

### 3.4. Blood Parameters

The blood test results (TG, TC, HDL-C, LDL-C, and FBG) are shown in Table 3. There were significant interactions between group and time in TG (*p* < 0.001) and LDL-C (*p* = 0.012), but not the others (all *p* > 0.05). The TG concentration decreased in the running group after 12 weeks of intervention (−0.27 ± 0.06 mmol/L, *p* < 0.001), and this decrease continued until the end of 24 weeks (−0.33 ± 0.06 mmol/L, *p* < 0.001). Although an increasing trend of LDL-C was found in the control group from baseline to 24 weeks (*p* < 0.05), there was a continual decline in the running group by −0.37 ± 0.18 mmol/L (*p* = 0.048) in 12-week and −0.46 ± 0.18 mmol/L (*p* = 0.020) at 24-week intervention. 

### 3.5. Cardiorespiratory Fitness

The interaction between group and time was found in maximal MET achieved in Bruce protocol, VO_2max_ (*p* < 0.001) and V_max_ (Table 4, *p* < 0.001). The simple main effect revealed that the maximal MET achieved in Bruce protocol was increased but PRE score was decreased across the training in the Tai-Chi and running groups (*p* < 0.05), but it remained similar across training times in the control group. The analysis also revealed that VO_2max_ (mL/kg/min) increased in both exercise intervention groups, while in the control group, the VO_2max_ remained unchanged throughout the 24-week study period, *p* < 0.001). The VO_2max_ significantly increased after the 12-week intervention with Tai-Chi group (5.4 ± 0.9 mL/kg/min, *p* < 0.001) and the running group (5.2 ± 0.8 mL/min/kg, *p* < 0.001) and continued to increase until the 24-week intervention (7.6 ± 1.1 mL/kg/min, *p* < 0.001 and 11.2 ± 0.9 mL/kg/min, *p* < 0.001, respectively). The changes in VO_2max_ in the running group were greater than in the Tai-Chi group after the 24-week intervention (*p* < 0.001).

In further analysis of interaction, V_max_ had increased by 0.8 ± 0.2 km/h (*p* = 0.001) and 1.4 ± 0.3 km/h (*p* < 0.001) in the Tai-Chi group and increased by 1.3 ± 0.2 km/h (*p* < 0.001) and 1.9 ± 0.3 km/h (*p* < 0.001) in the running group at 12 weeks and 24 weeks, respectively. There were significant differences in Vmax between the two exercise groups in the 12-week (*p* = 0.019), but not the 24-week, intervention (*p* > 0.05). Furthermore, HR_peak_ was found to increase from baseline by 14.8 ± 3.8 b/min in the Tai-Chi group and 9.5 ± 3.2 b/min in the running group after 24-week (Table 4).

## 4. Discussion

This study examined the effectiveness of Tai-Chi and running exercises on VO_2max_ and biomarkers in middle-aged adults after a 24-week supervised intervention. The findings from the current study revealed that both intervention groups can significantly improve overall cardiorespiratory fitness and biomarkers, including resting HR, DBP, TG, LDL-C, lean mass and MET achieved in Bruce protocol after 24-week supervised training. The current study controlled the exercise intensity (5 days/week and 60 min/day) and dietary intake for both the Tai-Chi and running groups. The total amount of exercise was based on the ACSM [6] and Federal Physical Activity Guidelines, which recommend at least 150 min of moderate-intensity exercise or 75 min of vigorous-intensity exercise per week to improve their cardiorespiratory fitness and health. Both exercise groups controlled their movement intensities at 66–74% of maximal heart rate, as this was pre-determined and averaged from all of the participants (all groups) based on the 50–60% of maximal VO_2max_ obtained in the Bruce graded exercise at the start of the experiments. This intensity level is considered to be moderate, which is designed for our participants who had no regular exercise habits and less than 1000 steps per day. For the Tai-Chi and running groups, movement intensity was supervised by instructors and participants were alerted by the haptic vibration feedback from the heart rate monitor during each exercise session. This arrangement was used to determine the objective comparison between exercise interventions. Compared to baseline, both the Tai-Chi and running groups increased their number of steps per day at 12 weeks and 24 weeks, while the no-exercise control group had a similar number of steps per day across training time. This confirmed that our exercise intervention was effective in promoting physical activity.

Both the Tai-Chi and running groups showed significantly lower resting heart rates than the no-exercise control at 24 weeks, but no differences between groups were found at baseline and 12 weeks. The reduced resting heart rates in both exercise groups could be due to the improvement in cardiorespiratory capacity as well as body and blood composition (lean mass, TG, HDL-C and LDL-C) after the 24-week supervised training. While the Tai-Chi group appeared to have a higher resting heart rate than the other two groups, further investigation with larger sample sizes should be considered to reduce the impact of the individual subject differences before a viable conclusion can be made.

Tai-Chi and running have different movement characteristics and their effects on cardiac function, peripheral blood circulation, proprioception and body control may also differ. The VO_2max_ of both intervention groups significantly increased over the intervention period; however, the increase in the running group was greater than in the Tai-Chi group after the 24-week intervention. The running exercise group appeared to induce more benefits related to cardiorespiratory fitness than the Tai-Chi group. One plausible explanation could be that running exercise mainly involves lower-limb bi-pedal movements, which is highly similar to the movement task used in cardiorespiratory tests (i.e., graded running test). This would activate more relevant motor units due to the increased efficient motor task transfer [23]. Another explanation is that running could be more effective to enhance cardiorespiratory capacity, as indicated by its better coordination with the pump function of the cardiovascular system, beneficial effects on pulmonary oxygen intake capacity, and improved muscle oxygen uptake [5,23]. While running involves both concentric and eccentric contractions, participants need to propel their body weight forward with repeated sole contacts with the ground. These characteristics would result in more efficient cardiac ejection and prevent the backflow of blood [5]. 

Tai-Chi is a relatively “easy to assess” approach to maintain physical performance. It is characterized as the movement of Yi and Qi, with Qi driven by Yi that is controlled by the mind. The movements of Tai-Chi are relatively slow and are controlled by the depth and frequency of respirations. Tai-Chi is suggested to improve muscular elasticity and breathing, which is regulated by several small muscles in the chest and abdominal wall. Since the movements of Tai-Chi are light and smooth, the stimulatory effects are relatively small and Tai-Chi results in steady improvements in VO_2max_. Although the improvements in cardiorespiratory fitness in Tai-Chi were less than in running exercises after 24-week supervised training in our study, Tai-Chi might promote dynamic balance and thus prevent falls, as shown in previous studies [24,25]. Moreover, it can enhance full-body muscle strength [25] and reduce mental stress through its meditation process [26,27]. Furthermore, Tai-Chi is suitable to be a home-based exercise that could be a new exercise alternative under COVID-19 situation.

Fat burning and lipolysis are slowly processed by implementing exercises with low and moderate intensities. Our findings indicated significant decreases in LDL-C and TG in the running group but a subtle decrease in the Tai-Chi group after the 24-week training. This is in line with the previous running and Tai-Chi studies [28,29]. Tsao et al. reported a decrease in blood lipids (TC, HDL-C and TG) in patients with hypertension problems in a 12-week aerobic exercise intervention, but no obvious change in blood lipids within the first four weeks. On the other hand, the Tai-Chi study [29] did not find significant changes in body composition and lipids in Chinese older adults after the 12-month Tai-Chi training. One would argue that aerobic exercise can influence the number and size of the lipoprotein particles, resulting in faster fat metabolism and low-density lipoprotein decomposition as well as reduce LDL cholesterol transport to the arterial wall. While all groups were reminded to keep dietary intake and activity level constant over the 24 weeks, both the Tai-Chi and running groups demonstrated higher dietary intakes in all carbohydrates, proteins and fats over training time, but remained unchanged in the control group, implying that appetite is triggered by exercises. The increased LDL-C in the control group could be related to the prolonged sedentary lifestyle over the 24-week study period, which is in line with the findings from a previous study showing that higher LDL-C levels are related with sedentary behavior [29]. These findings provide further support that moderate intensity or exercise habit is necessary for the sedentary middle-aged population.

Interestingly, the results from this study indicated both Tai-Chi and running exercise effectively reduced blood pressure (DBP) and increased lean mass at the end of 24-week intervention. Tai-Chi was proved to reduce blood pressure and strengthen both the systolic and diastolic functions of the cardiovascular systems, as similarly suggested in other aerobic exercises [30,31]. Blood pressure is influenced by both blood rheology and viscous shear stress. In addition, Pitsavos et al. [32] revealed that 16-week aerobic exercise decreased systolic and diastolic blood pressure in patients with hypertension. A recent meta-analysis [33] found that brisk walking (increased walking speed) can improve VO_2max_ and lean mass as well as decrease body mass, BMI and resting diastolic blood pressure, which are considered as the risk factors for cardiovascular diseases. Together with the current findings, regular exercises at moderate intensity would improve diastolic velocity and allow full cardiac ejection [34,35,36].

Cardiorespiratory fitness is one of the predictors of cardiovascular disease and other chronic diseases [1,2]. Other risk factors include genetic, sex, age, oxygen transport system, muscle oxygen uptake, and the exercise types can influence VO_2max_ level. There is a dose–response relationship between exercise and cardiorespiratory fitness [37,38,39]. In the current study, VO_2max_ increased from baseline in both groups at 12 weeks and this improvement continued until the 24-week intervention. However, running exercises showed larger improvement in VO_2max_ than Tai-Chi exercises after the 24-week intervention. While cardiac output is the key limiting factor to increase VO_2max_, it is associated with exercise intensity. After the 24-week intervention, both V_max_ and peak heart rate were also increased during the graded exercise test, indicating a higher graded exercise performance (higher MET achieved in Bruce protocol and lower RPE score at 50–60% VO_2max_) for both the Tai-Chi and running exercise groups. The combination of these biomarkers could help to prevent cardiac stroke, keep stroke volume stable, and allow maximal oxygen uptake during the increase in physical activities.

When interpreting our results, some limitations should be considered. First, only middle-aged Chinese males were recruited in this study. The results may not be generalizable to female and Western participants. Gender differences may potentially show different physical activity habits, body compositions and metabolic parameters [40], and cardiovascular risk factors over 45 years of age and 55 years of age for males and females, respectively. Second, there was a small sample size for this study, as our study samples were recruited in the same city, which could be another limitation of this study. Although we provided a monetary incentive, supervised training and a training diary, some of the participants dropped out and failed to complete all exercise sessions (a total of 120 sessions). In the future, implementing virtual reality technology into Tai-Chi, running and other physical activities should be considered for longer training durations and larger sample sizes. Third, although we controlled and monitored the training duration and intensity in our study, other confounding factors in their normal daily life (e.g., food intake, physical activity) could influence metabolic outcomes.

## 5. Conclusions

Both Tai-Chi and running exercises with moderate intensity can effectively reduce resting heart rate, diastolic blood pressure and cardiorespiratory fitness in sedentary middle-aged adults after 24-week supervised training. Increased lean mass, reduced blood pressure, and improved blood composition and VO_2max_ in both exercise groups suggest that Tai-Chi can be used as an alternative exercise to running for improving physical health in sedentary middle-aged adults.

## Figures and Tables

**Table 1:** Demographic characteristics of the participants by exercise groups.

	Tai Chi (*n* = 10)	Running (*n* = 14)	Control (*n* = 12)
Age (year)	57.1 ± 3.8	56.8 ± 6.5	53.1 ± 4.9
Height (cm)	165.9 ± 4.5	166.9 ± 7.1	162.7 ± 5.0
Body mass (kg)	65.2 ± 5.1	66.7 ± 8.6	61.3 ± 7.6
Lean mass (kg)	50.9 ± 3.9	52.0 ± 6.8	47.9 ± 5.9
BMI (kg/m^2^)	23.7 ± 2.0	23.8 ± 1.9	23.1 ± 2.3
Average number of step/day (step)	763.7 ± 102.5	766.7 ± 144.5	829.7 ± 121.5
Self-reported physical activity (MET-min/week) *	1347.1 ± 339.0	1537.6 ± 288.1	1354.1 ± 307.3

BMI refers to Body Mass Index, which is derived as the body mass divided by the square of the body height; MET refers to Metabolic Equivalent of Task, which is the measure of ratio of the rate at which a person expends energy. * based on International Physical Activity Questionnaire (ACSM 2010).

**Table 2:** Physical activity and nutrient uptake among Tai-Chi (*n* = 10), running (*n* = 14) and control (*n* = 12) groups at baseline, 12-week and 24-week intervention (Mean ± SD).

	Time (wk)	Tai-Chi (T)	Running (R)	Control (C)	T vs. C *p* Value	R vs. C *p* Value	T vs. R *p* Value	Group * Time *p* Value
Average number of steps/day (Step)	0	763.7 ± 102.5	766.7 ± 144.5	829.7 ± 121.5	0.695	0.643	1.000	**<0.001**
	12	1734.6 ± 102.4 **	1671.5 ± 107.6 **	707.6 ± 153.6	**<0.001**	**<0.001**	0.537	
	24	1563.7 ± 102.5 **	1566.7 ± 144.5 **	774.8 ± 127.9	**<0.001**	**<0.001**	1.000	
Self-reported physical activity (MET-min/week)	0	1347.1 ± 339.0	1537.6 ± 288.1	1354.1 ± 307.3	0.161	1.000	0.055	**<0.001.**
	12	1340.6 ± 339.0	1539.9 ± 288.2	1558.6 ± 307.8 **	0.328	1.000	0.388	
	24	1342.9 ± 339.0	1542.1 ± 288.2	1560.8 ± 307.8 **	0.341	0.998	0.372	
Reported dietary intake (kcal)	0	2703.0 ± 1.6	2702.6.6 ± 1.5	2702.7 ± 1.5	0.968	0.993	0.890	**<0.001**
	12	2729.9 ± 3.2 *	2716.7 ± 13.8 **	2702.9 ± 1.5	**<0.001**	**0.007**	0.011	
	24	2762.5 ± 21.7 ** ^†^	2768.6 ± 5.8 ** ^††^	2703.0 ± 1.5	**<0.001**	**<0.001**	0.773	
Carbohydrate intake (g/day)	0	354.4 ± 5.3	357.4 ± 4.7	353.6 ± 5.3	0.973	0.174	0.410	**0.013**
	12	368.2 ± 4.3 **	367.6 ± 4.2 **	354.4 ± 8.7	**<0.001**	**0.001**	0.981	
	24	361.2 ± 4.9 ^†^	364.1 ± 4.2 *	355.6 ± 8.2	0.163	**0.015**	0.381	
Fat intake (g/day)	0	94.0 ± 1.4	94.8 ± 1.3	93.8 ± 1.4	0.973	0.174	0.410	**0.007**
	12	97.6 ± 1.2 **	97.5 ± 1.1 **	94.0 ± 2.3	**0.001**	**0.001**	0.981	
	24	96.1 ± 1.3 *	96.8 ± 1.1 *	94.9 ± 1.3	0.120	0.002	0.382	
Protein intake (g/day)	0	89.9 ± 1.4	90.7 ± 1.3	89.7 ± 1.4	0.973	0.174	0.410	**0.007**
	12	91.6 ± 1.2 *	91.5 ± 1.1	89.9 ± 2.3	0.106	0.138	0.981	
	24	91.1 ± 1.3	91.8 ± 1.1	90.2 ± 1.4	0.374	**0.011**	0.382	

Notes: Bolded denotes significance at *p* < 0.05. * and ** significantly different compared to the baseline within group, *p* < 0.05 and *p* < 0.01, respectively. ^†^ and ^††^ significantly different between 12 weeks and 24 weeks within groups, *p* < 0.05 and *p* < 0.01, respectively.

**Table 3:** Resting heart rate (HR), blood pressure and blood parameters among Tai-Chi (*n* = 10), running (*n* = 14) and control (*n* = 12) groups at baseline, 12-week and 24-week intervention (Mean ± SD).

	Time (wk)	Tai-Chi (T)	Running (R)	Control (C)	T vs. C *p* Value	R vs. C *p* Value	T vs. R *p* Value	Group * Time *p* Value
Resting heart rate (b/min)	0	72.9 ± 10.87	69.0 ± 6.5	68.1 ± 6.4	0.146	0.679	0.068	**0.057**
	12	67.0 ± 7.4	66.2 ± 5.7	69.6 ± 7.1	0.109	0.175	0.570	
	24	66.2 ± 8.0 **	65.3 ± 5.8 **	72.2 ± 8.4	**0.014**	**0.018**	0.528	
Body mass (kg)	0	65.2 ± 5.1	66.7 ± 8.6	61.3 ± 7.6	0.710	0.232	1.000	**<0.001**
	12	65.7 ± 5.2	67.2 ± 8.7	61.8 ± 7.7	0.714	0.235	1.000	
	24	64.8 ± 5.1	66.3 ± 8.6	61.9 ± 7.6	1.000	0.443	1.000	
Lean mass (kg)	0	50.9 ± 3.9	52.0 ± 6.7	47.9 ± 5.9	0.709	0.234	1.000	**<0.001**
	12	53.9 ± 4.2	55.1 ± 7.1	48.5 ± 6.0	0.143	**0.028**	1.000	
	24	56.4 ± 7.1	51.3 ± 8.1	43.4 ± 5.4	**<0.001**	**<0.001**	1.000	
Systolic blood pressure (SBP, mmHg)	0	124.7 ± 3.2	126.1 ± 8.5	126.8 ± 11.1	0.643	0.851	0.750	0.703
	12	119.7 ± 5.6	124.6 ± 12.8	126.8 ± 10.6	0.217	0.639	0.376	
	24	116.8 ± 6.1	121.8 ± 8.8	125.3 ± 11.5	0.095	0.388	0.307	
Diastolic blood pressure (DBP, mmHg)	0	77.7 ± 6.9	78.2 ± 5.8	79.6 ± 5.9	0.544	0.597	0.852	**0.006**
	12	72.0 ± 8.0	72.9 ± 7.6	83.5 ± 8.6	**0.010**	**0.004**	0.818	
	24	70.2 ± 7.7 **	70.2 ± 5.6 ** ^†^	83.6 ± 7.9	**0.001**	**<0.001**	0.985	
Total cholesterol (TC, mmol/L)	0	4.33 ± 0.54	4.35 ± 0.64	4.40 ± 0.56	0.787	0.842	0.929	0.073
	12	4.24 ± 0.48	4.11 ± 0.60	4.37 ± 0.61	0.591	0.246	0.579	
	24	4.04 ± 0.58	4.00 ± 0.62	4.46 ± 0.59	0.115	0.058	0847	
Triglycerides (TG, mmol/L)	0	1.49 ± 0.19	1.51 ± 0.21	1.49 ± 0.33	0.976	0.884	0.865	**<0.001**
	12	1.36 ± 0.22	1.24 ± 0.23 **	1.53 ± 0.32	0.141	**0.008**	0.276	
	24	1.37 ± 0.22	1.18 ± 0.22 ** ^†^	1.56 ± 0.32	0.103	**0.001**	0.074	
High-density lipoprotein cholesterol (HDL-C, mmol/L)	0	1.36 ± 0.33	1.34 ± 0.29	1.34 ± 0.23	0.897	0.940	0.838	0.694
	12	1.41 ± 0.26	1.42 ± 0.24	1.35 ± 0.22	0.574	0.444	0.882	
	24	1.49 ± 0.26†	1.46 ± 0.27	1.38 ± 0.24	0.329	0.444	0.775	
Low-density lipoprotein cholesterol (LDL-C, mmol/L)	0	2.63 ± 0.54	2.63 ± 0.52	2.68 ± 0.50	0.801	0.790	0.992	**0.012**
	12	2.46 ± 0.51	2.25 ± 0.48 **	3.10 ± 0.96 *	**0.046**	**0.005**	0.491	
	24	2.41 ± 0.50	2.18 ± 0.44 ** ^††^	3.12 ± 0.97 *	**0.028**	**0.002**	0.443	
Fasting blood glucose (FBG, mmol/L)	0	5.37 ± 0.21	5.38 ± 0.21	5.38 ± 0.25	0.881	0.942	0.933	0.088
	12	5.40 ± 0.12	5.30 ± 0.20	5.37 ± 0.27	0.773	0.410	0.286	
	24	5.19 ± 0.16	5.16 ± 0.22	5.32 ± 0.25	0.197	0.089	0.753	

Notes: Bolded denotes significance at *p* < 0.05. * and ** are significantly different compared to the baseline within group, *p* < 0.05 and *p* < 0.01, respectively. ^†^ and ^††^ significantly different between 12 weeks and 24 weeks within group, *p* < 0.05 and *p* < 0.01, respectively.

**Table 4:** Maximal oxygen uptake, maximal aerobic speed and peak heart rate among Tai-Chi (*n* = 10), running (*n* = 14) and control (*n* = 12) groups at baseline, 12-week and 24-week intervention (mean ± SD).

	Time (wk)	Tai-Chi (T)	Running (R)	Control (C)	T vs. C *p* Value	R vs. C *p* Value	T vs. R *p* Value	Group * Time *p* Value
Maximal MET achieved in Bruce test (MET-min/week)	0	9.2 ± 1.0	9.6 ± 1.0	9.2 ± 0.9	1.000	0.925	1.000	**<0.001**
	12	10.9 ± 0.8	11.0 ± 1.0	9.1 ± 1.0	**<0.001**	**<0.001**	1.000	
	24	11.7 ± 0.9	12.8 ± 0.8	9.4 ± 1.0	**<0.001**	**<0.001**	**0.019**	
Rating of Perceived Exertion at 50–60% VO_2max_ (RPE)	0	12.5 ± 0.5	12.3 ± 0.5	12.3 ± 0.5	0.830	0.992	0.669	**<0.001**
	12	9.5 ± 0.5 *	9.6 ± 0.5 *	12.3 ± 0.5	**<0.001**	**<0.001**	0.876	
	24	9.4 ± 0.5 *	9.3 ± 0.5 **	12.3 ± 0.5	**<0.001**	**<0.001**	0.924	
Maximal oxygen uptake (VO_2max,_ mL/kg/min)	0	32.6 ± 3.5	33.4 ± 3.3	31.1 ± 3.4	0.305	0.089	0.305	**<0.001**
	12	38.0 ± 3.2 **	38.6 ± 3.3 **	30.6 ± 2.2	**<0.001**	**<0.001**	0.603	
	24	40.2 ± 2.1 ** ^††^	44.6 ± 2.8** ^††^	31.4 ± 2.2	**<0.001**	**<0.001**	**<0.001**	
Maximal aerobic speed (V_max_, km/h)	0	5.7 ± 0.5	5.9 ± 0.8	5.9 ± 0.8	0.601	0.919	0.524	**<0.001**
	12	6.5 ± 0.5 **	7.2 ± 0.6 **	6.1 ± 0.9	0.208	**<0.001**	**0.019**	
	24	7.1 ± 1.1 **	7.9 ± 1.0 ** ^†^	6.1 ± 0.9	**0.020**	**<0.001**	0.085	
Peak heart rate (HR_peak_, b/min)	0	166.8 ± 9.9	166.8 ± 15.2	164.9 ± 13.6	0.745	0.725	0.998	0.440
	12	176.7 ± 14.7	172.7 ± 12.2	168.8 ± 12.6	0.164	0.445	0.465	
	24	181.6 ± 11.4 **	176.3 ± 6.8 **	167.2 ± 13.5	**0.003**	**0.038**	0.239	

Notes: Bolded denotes significance at *p* < 0.05. * and ** significantly different compared to the baseline within groups, *p* < 0.05 and *p* < 0.01, respectively. ^†^ and ^††^ significantly different between 12 weeks and 24 weeks within groups, *p* < 0.05 and *p* < 0.01, respectively.

## Data Availability

The data can be obtained from the corresponding authors upon reasonable request.
